# Abstracts from German Journals

**Published:** 1872-05

**Authors:** Ch. Raushenberg

**Affiliations:** Atlanta, Georgia


### ABSTRACTS FROM GERMAN JOURNALS.

BY CH. RAUSHENBERG, M.D., ATLANTA, GEORGIA.

[Read Before the Atlanta Academy of Medicine.]

*ON THE TREATMENT OF VARIX BY SUBCUTANEOUS INJECTIONS OF ERGOTINE. By Dr. PAUL VOGT, Assistant Physician to the Surgical Polyclinic at the University of Greifswalde.*

After the observations of Von Langenbeck in relation to the cure of two aneurisms by subcutaneous injections of ergotine (*Berliner Klinische Wochenscrift*, 1869, No. 12, p. 117)—followed, first, by a communication of Schneider, made at the meeting of the Society for Scientic Medicine, at Koenigsberg, on the 25th of May, 1869, on the cure of an aneurism of the femoral artery, and finally by one from Dutoit (in *Von Langenbeck's Archives*, Vol. xii.,) on the cure of one of the subclavian artery by the same means—had almost beyond a doubt established the effect of the preparations of ergot on the blood-vessels, particularly in the form of subcutaneous injections of ergotine, as they were first used by Drasche, of Vienna, against hæmoptysis (*Central-blatt fuer Medicin Wissenchaften*, 1868, No. 52), I commenced last summer a series of experiments in relation to the effect of ergotine in other morbid conditions of blood-vessels.

I first tested its effect in a disease in which our surgical remedies, as far as a radical cure is concerned, have been almost entirely powerless, to wit—*in varicose veins of the leg.* As the leading principle which so far has governed the radical treatment of varices—the production of more or less extensive obliteration of the cavity of the diseased vein—by whatever method it may be carried into effect, involves dangers much greater than those resulting from the disease itself, we have generally, by our efforts to use only safe methods of relief, been confined to a mere palliative treatment.

The effect of ergotine in this disease was tested with a view of regulating the circulation, and removing expansion and engorgement of the vessels.

A man sixty years old, who had suffered for years from extensive varicose veins of the right leg, was treated with subcutaneous injections of extract secalis cornut aqu 2, 0 ( ℨ i.) with spirit vini and glycerine ana 7, 5 (108.½ grs.), in such a manner that principally two localities, where the varices were most largely developed, were used for injections. The outlines of a varix about six centimetres (nearly two inches) long, and as thick as the little finger of the human hand, running across the tibia, were marked on the surface of the skin by lunar caustic, and about its center a whole syringe of solution of ergotine (0.12 or 1 4-5 grs.) was injected nnder the skin every other day. In the course of eight days, the varix had totally disappeared, and after the patient had been walking about six weeks, in his usual way, no sign or symptom of the old disease could be seen or felt on the place where it existed before.

Another varix, of the size of a hazel-nut, on the external calf of the leg, was made to disappear in the same individual, by one single ergotine injection under the skin above it.

At the place where the injections had been made, a hard and rather circumscribed infiltration made its appearance, but showed, notwithstanding the thin and atrophic skin of the rather senile individual, no inclination to inflame or become gangrenous, but disappeared without causing any notable pain or inconvenience.

For the purpose of becoming still better acquainted with the effect of ergotine on the blood-vessels, I caused Dr. Potel to institute two experiments, with the intention of—

1. Testing microscopically the effect of ergotine on the vessels of the mesentery of the frog.

2. Establishing sphygmographically the changes of the pulse produced by ergotine.

These experiments were made under the supervision of Prof. Landois*, and established the fact, microscopically and syphygmographically, that ergotine acts on the muscular coat of the blood-vessels, the arteries as well as the veins (Inaugural Dissertation, Griefswalde, 1871.)

After this, numerous patients in the surgical clinic and polyclinic of this city, with varicose veins, were treated by subcutaneous injections of ergotine, in the manner above described, and the astonishingly favorable effect of this treatment, even in the most enormous varicose enlargements, was distinctly confirmed. For an explanation of this effect, we must take the following points into consideration:

1. The above experiments and clinical observations demonstrate that the subcutaneous injections of ergotine cause, primarily, a contraction of the muscular coat of the arteries. By this diminution of the calibre of the arteries, which most prominently takes place in the medium-sized vessels, as they are most richly supplied with smooth muscular fibres, the quantity

*The author of a late work, "Die Lehre vom Arterien Pulse," (Teachings on the Arterial Pulse)—full of original investigations and important physiological facts.—TRANSLATOR.

of the blood which reaches the veins is diminished, but the velocity of its motion increased.

2. The ergotine causes also a contraction of the muscular coat of the veins; and if in varices of very long standing this faculty of contraction may almost seem to be abolished, it still exists, to more or less extent, in many places along the course of the diseased vessels; and that it is very considerable in varices of not too long duration, is demonstrated by the fact that the enormous varices which we frequently see in pregnant women are often, within a few days after delivery, reduced to very common varicose enlargements.

Whether this favorable effect—which, so far, has been clearly established in all cases—will finally prove sufficient for a radical cure, can only be demonstrated by a longer observation and control of the cases treated. The results, however, so far obtained, invite at least further experiment and further application of a remedy which, in all cases, can be used without any danger; and I have already commenced to use it, and partly with favorable results, in other venous diseases — for instance, varicocele, hemorrhoids, and certain forms of angioma.

In order to gain further physiological clues for the therapeutical use of this agent, I endeavored to determine whether the subcutaneous ergotine injections would first and immediately produce contraction of the vessels at the place where they are made, or *whether this effect took place, only after absorption, from the center of vaso-motor action.* The fundamental experiments were the following.

1. Ergotine 0, 12 (1.4-5 grs.) was subcutaneously injected into the right femur of a frog, and, two hours and a half after, this leg amputated below the knee. Hemorrhage very small, while amputation of the leg of the other side, at the same place and at the same time, shows considerable hemorrhage. The same experiment, repeated twice on other frogs, shows very little difference in the hemorrhage—giving rise to the idea, that perhaps the compression caused by the injected fluid occasioned the difference observed in the first experiment.

2. The cervical portion of the sympathetic nerve of a rabbit is divided on both sides as high up as the upper cervical ganglion. After the vascular congestion and increased temperature following this operation, on both sides of the head, is well established, 0, 06 (9-10 grs.) ergotine is subcutaneously injected at the base of the right ear. No material difference between the two ears can be noticed within from one-half an hour after the injection up to the next day.

3. After injecting at the base of the right ear of a rabbit 0, 10 (1½ grs.) ergotine, the cervical portion of the sympathetic nerve of the same side, as far as the upper cervical ganglion, is divided. Soon afterwards, a very considerable engorgement of the vessels of that ear takes place; but the ear on the other side, where the sympathetic nerve has also been divided without previous injection of ergotine, shows the same increased vascularity as the ear of the other side.

After all this, we must arrive at the following resume: That ergotine, subcutaneously injected, causes contraction of the muscular coat of the blood-vessels; that this contraction of the smooth muscular fibres is caused by the intermediation of the vaso-motor centers. Whenever the influence of the latter is interrupted, the effect of ergotine is paralyzed. If ergotine affects the vessels at the place where it is injected, with any more intensity than on more distant portions of the body, it may be caused by a direct influence on the peripheric extremities of the sympathetic nerve, analogous to the local effect of the subcutaneous injections of morphine, which, besides their general effect, seem to produce, also, a local anæsthesia of the peripheric nerves of the locality where the injection is made.—*Berliner Klinische Wochenscrift, March,* 1872, *No.* 10.

---

### *APOMORPHINE—A NEW REMEDY.*

It appears that the *Materia Medica* is about to be enriched by an important remedy—apomorphine, an emetic apparently superior to all which have been used before. Two published investigations about the physiological effects of this remedy are before us: one by V. Siebert, of Dorpat ("Investigations on the Physiological Effect of Apomorphine"—*Archives fuer Heil-*

*kunde*, xii., 6), and another one by Riegel and Boehm ("On the Emetic Effect of Apomorphine"—*Deutches Archives fuer Klinische Medicine*, ix., 2.)

After it had already been prepared, in 1845, by Asppe and other chemists, Matthiesen and Wright, in England, have lately again, by treating morphine with hydrochloric acid, produced it as a hydrochloric salt, the base of which (apomorphine) has simply originated out of morphine by the escape of water, to wit: $C_{17}H_{19}NO_3$ (morphine)—$H_2 0=C_{17}H_{17}NO_2$ (apomorphine.) The same chemists have given notice of its emetic effect.

Numerous experiments on animals and human beings have taught that apomorphine is a reliable and speedy emetic (acting within from four to sixteen minutes, according to Riegel and Boehm), which has no very disagreeable concomitant effects of any kind, but the great advantage of being well suited for subcutaneous injections—a quality not belonging to any other known emetic, and one of great importance in the treatment of children, lunatics, unconscious patients, and other cases. Slight vertigo, heaviness of the head, inclination to yawn, and præcordial uneasiness of very short duration, are the only symptoms which so far have been observed to occur when it is administered, but they disappear as soon as emesis sets in. After effects on the intestinal canal alike those of tartar emetic, or inflammation and suppuration at the point of injection, have never been observed. Its physiological effects on the pulse, temperature of the skin, etc., are of no practical importance. The quantity necessary for this effect by hypodermic injection is, in the human being, according to Siebert, 0,006 to 0,007 (about 1-10 grs.), and oscillates, according to Riegel and Boehm, between 0,03 and 0,04 (about $\frac{1}{2}$ to $\frac{2}{3}$ of a grain). The latter used a solution containing one per cent. for their preparations.

This preparation has principally been obtained from England (under the name of hydrochlorate of amorphia, from McFarlan & Co., Royal Medical Warehouse, 17 North Bridge, Edinburgh), as a pale, greenish-gray powder.—*Berliner Wochenschrift, January*, 1872, *No.* 5.

